# Carotid plaques and neurological impairment in patients with acute cerebral infarction

**DOI:** 10.1371/journal.pone.0226961

**Published:** 2020-01-03

**Authors:** Tongtian Ni, Yi Fu, Wei Zhou, Min Chen, Jianwei Shao, Weijun Zhou, Enqiang Mao, Erzhen Chen

**Affiliations:** 1 Department of Emergency Medicine, Rui Jin Hospital, School of Medicine, Shanghai Jiao Tong University, Shanghai, China; 2 Department of Neurology & Institute of Neurology, Rui Jin Hospital, School of Medicine, Shanghai Jiao Tong University, Shanghai, China; 3 Department of Ultrasound, Rui Jin Hospital, School of Medicine, Shanghai Jiao Tong University, Shanghai, China; Weill Cornell Medicine-Qatar, QATAR

## Abstract

**Objective:**

To determine whether the coexistence of carotid atherosclerosis plaque affects the neurological function of cerebral infarction.

**Methods:**

A total of 1078 patients with acute cerebral infarction were enrolled, all patients were divided into carotid plaque group (*n* = 702) and non-carotid plaque group (*n* = 376). Meanwhile, all patients were divided into mild group (*n* = 624) and moderate to severe group (*n* = 454). The difference of the incidence of carotid plaque between the mild and moderate to severe group was analyzed.

**Results:**

In the 1078 patients with cerebral infarction, the NIHSS score in the carotid plaque group was significantly higher than that in the non-carotid plaque group (*P*<0.05). The number of mild cases without carotid artery plaque group was larger than that of plaque group (*P*<0.05), and the number of moderate to severe cases in carotid plaque group was larger than that in non-plaque group (*P*<0.05). In patients with carotid atherosclerotic plaque, the risk of moderate to severe cerebral infarction was 2.11 times higher than that without carotid artery plaque. Lastly, patients with single plaques were 1.82 times more likely to develop moderate to severe cerebral infarction than those without carotid plaque, while patients with multiple carotid plaques were 2.41 times higher to get moderate or severe cerebral infarction than those without carotid plaque.

**Conclusions:**

The incidence of carotid atherosclerotic plaques may be related to neurological deficits in patients with acute cerebral infarction.

## Introduction

Stroke, with high morbidity, mortality and disability rates, is one of the leading causes of death in the world [1–3]. Cerebral infarction which even accounts for up to 80% of the whole stroke patients in China, is the most common type of stroke [2,4]. Our previous epidemiological studies in Shanghai RUIJIN Hospital also found that cerebral infarction is one of the common diseases in our emergency [5]. It is of utmost importance to select patients with high risk of negative outcomes and to address them through a more intensive pharmacological and rehabilitative approach[6]. Therefore, it is of great medical value to define the risk factors related to the cerebral infarction [7].

Atherosclerosis is considered as the most important pathological factor for stroke in developed countries [3]. Several prospective study[8–11]found that carotid intima-media thickness (IMT) could independently predict future vascular events. Research has also demonstrated that atherosclerotic plaque may play a role in risk of cerebrovascular accidents [10,12–15]. A number of clinical studies have confirmed that there is a causal relationship between plaque and cerebral infarction [10,15–19]. Cerebral infarction can be prevented by treating the carotid atherosclerotic stenosis [13,20,21]. Therefore, the severity of carotid stenosis caused by carotid atherosclerotic plaque has been widely used as an imaging indicator of stroke risk, and is a key indicator of atherosclerotic disease treatment [22–24]. However, up to now, few studies have been published on the correlation between carotid atherosclerotic plaques and the neurological function of cerebral infarction and the clinical implications of this coexistence.

As a cheap, noninvasive and non-ionizing imaging technology, examination of plaques using carotid ultrasonography is currently the only real-time imaging method that can observe the shape, hemodynamic and elastic characteristics of arterial wall[25]. Therefore, carotid ultrasonography is an ideal tool for long-term cardiovascular disease risk assessment for the asymptomatic patients, helping refine the risk score stratification and guiding intervention to the patients [16,26–29].

Hence, this study was used to determine whether the coexistence of carotid atherosclerosis plaque affected the neurological function of cerebral infarction, and to provide a reliable clinical basis for early intervention of cerebral infarction.

## Materials and methods

### Study subjects

All patients with the first onset of cerebral infarction were included in the current study from Jan 2013 to December 2017, at Ruijin Hospital affiliated to Shanghai Jiaotong University School of Medicine, Minhang Hospital affiliated to Fudan University, and Public Hospital of Shanghai Pudong District. All patients were based on 48h of onset of clinical manifestation and verification of ischemic lesions by cranial CT or MRI imaging. The exclusion criteria were cerebral hemorrhage, transient ischemic attack, cerebral vascular malformation, aneurysms, other intracranial lesions, coronary heart disease, peripheral vascular disease, tumor, immunological disorders, blood diseases, infection, severe kidney and liver dysfunction, and other metabolic disorders such as thyroid dysfunction. This study was approved by Ruijin Hospital Ethics Committee and Shanghai JiaoTong University School of Medicine (201112). Written informed consent was obtained from each patient or their family members. All data were fully anonymized.

### Clinical variables

The demographic data concerning gender, age, and risk factors (hypertension, hyperlipidemia, and diabetes were obtained for each patient. Hypertension was defined as systolic blood pressure (SBP) greater ≥140 mmHg and/or diastolic blood pressure (DBP) greater ≥90 mmHg, or treatment with antihypertensive medication. Diabetes mellitus was defined as a fasting plasma glucose level of ≥126 mg/dL, a random plasma glucose level of ≥200 mg/dL, an HbA1c level of ≥6.5%, or taking anti-diabetic medication Hyperlipoidemia was diagnosed as plasma total cholesterol (TC) ≥ 220 mg/dL or total triglyceride (TG) ≥ 150 mg/dL or low-density lipoprotein cholesterol (LDL-C) ≥ 140 mg/dL and/or current use of a lipid-lowering drug. Smoking status of the patients was defined as smokers and nonsmokers, and the status were recorded according to patients self-provided.

Patients with cerebral infarction were treated according to the guidelines for the diagnosis and treatment of cerebral infarction[30]. All blood pressure statuses and complete clinical data were recorded, and electrocardiography, chest radiography, CT or MR imaging were performed.

### Evaluation of neurological impairment of the stroke

All patients were examined immediately after admission by a neurologist. Neurological function of patients was assessed with the National Institutes of Health Stroke Scale (NIHSS) [31]at onset of stroke. A total of 1078 patients with cerebral infarction were included, which categorized into two groups according to the NIHSS score: mild group (patients with baseline NIHSS ≤ 3, they would be best suited to the definition of “minor stroke” n = 624)[32], moderate to severe group (patients with baseline NIHSS > 3, n = 454).

### Biochemical analysis

Patient blood samples were collected within 48 h following acute cerebral infarction and stored at -80°C until analysis. TG, TC, high-density lipoprotein cholesterol (HDL-C), LDL-C, and blood glucose were measured using standard methods at the Clinical Laboratory Department of Ruijin Hospital.

### Duplex ultrasonography protocol

All cerebral infarction patients within one week after admission were examined by extracranial carotid artery ultrasound, which was performed using ESAOTE MyLab 90 and Philips IU22 system. Both vertical and transverse scanning along the lateral edge of the sternocleidomastoid muscle were performed while the cerebral infarction patients lay in supine position, with his head turned to the opposite side. Carotid plaques were defined as focal echogenic thickenings with a minimal intimal plus medial thickness ≥ 1.2 mm[8,33]. The patients were divided into the carotid plaque group and non-plaque group according to the existence of a carotid plaque after the ultrasound findings. The study included 702 patients with plaque and 376 patients without plaque.

### Statistical analysis

This study is a cross-section study. The statistical analysis of the data was performed with SPSS 19.0 software. The enumeration data were presented as the means($\bar{x}$*)* ± *standard deviation (SD)*. Independent samples t-test was used to compare the mean values of subgroup analysis includes the plaque group and the non-plaque group or mild group and moderate to severe group, whereas χ2 test was used to compare proportions.

Binary logistic regression models were used for calculating odds ratios (OR), 95% confidence interval and corresponding *P* values between the cerebrovascular factors and the risk of plaque. Binary logistic regression models were also used for calculating odds ratios (OR), 95% confidence interval and corresponding *P* values between the cerebrovascular factors and plaque and the risk of moderate to severe cerebral infarction. Multivariable logistic regression models were used to determine the association (OR and 95% confidence interval) between plaque number and risk of moderate to severe cerebral infarction. *P* < 0.05 was considered statistically significant.

## Results

### Clinical characteristics of the patients

A total of 1078 patients with acute cerebral infarction were enrolled, aged 37 to 86 years old, of which 719 were male (66.70%), female 359 cases (33.40%), the average age was 58.91±8.25 years, of which 254 (23.56%) cases with diabetes and 686 (63.64%) cases with a history of hypertension.

### Comparison of clinical and laboratory data between carotid plaque and non-carotid plaque group

Significant differences were found in age, and several known cerebrovascular risk factors such as history of hypertension and diabetes, and LDL-C, blood glucose, systolic blood pressure (SBP), and NIHSS score between the carotid plaque and non-carotid plaque groups of cerebral infarction patients (*P* < 0.05). (Table 1)

**Table 1:** Comparison of clinical and laboratory data between carotid plaque and non-carotid plaque group (n%; $\bar{x}$ ± SD).

	Plaque (+) (n = 702)	Plaque (-) (n = 376)	*P* value
**Mean age(years)**	60.05±7.06	56.80±9.77	<0.001*
**Gender (male%)**	479(68.23)	240(63.83)	0.144
**Hypertension (%)**	477(67.95)	209(55.59)	<0.001*
**Diabetes mellitus (%)**	194(27.64)	60(15.96)	<0.001*
**Hyperlipoidemia (%)**	366(52.14)	164(43.62)	0.008*
**Risk factors at admission**			
**TG (mmol/L)**	1.94±1.29	1.81±1.17	0.093
**TC (mmol/L)**	4.63±1.30	4.61±2.92	0.904
**HDL-C (mmol/L)**	1.09±0.34	1.10±0.37	0.628
**LDL-C (mmol/L)**	3.00±0.99	2.82±0.92	0.003*
**Blood glucose (mmol/L)**	6.42±2.80	5.97±3.23	0.018*
**SBP(mmHg)**	145.21±19.55	140.61±21.62	<0.001*
**DBP(mmHg)**	82.54±11.14	83.43±12.06	0.222
**NHISS**	4.39±4.17	3.11±3.99	<0.001*

Plaque (+): carotid plaque group, plaque (−): non-carotid plaque group, TG: total triglyceride, TC: total cholesterol, LDL-C: low-density lipoprotein cholesterol, HDL-C: high-density lipoprotein cholesterol, SBP: systolic blood pressure, DBP: diastolic blood pressure.

*P<0.05 was considered statistically significant.

### Comparison of clinical and laboratory data between mild and moderate to severe groups

A history of diabetes, blood glucose and SBP was found significant difference between mild and moderate to severe groups (*P* < 0.05). (Table 2).

**Table 2:** Comparison of clinical and laboratory data between mild and moderate to severe groups (n%; $\bar{x}$±SD).

	Mild Group (n = 624)	Moderate to severe Group (n = 454)	*P* value
**Mean age(years)**	58.79±8.39	59.08±8.06	0.567
**Gender (male%)**	415(66.51)	304(66.96)	0.876
**Hypertension (%)**	384(61.54)	302(66.52)	0.093
**Diabetes mellitus (%)**	129(20.67)	125(27.53)	0.009*
**Hyperlipoidemia (%)**	318(50.96)	212(46.70)	0.167
**Risk factors at admission**			
**TG (mmol/L)**	1.91±1.23	1.87±1.28	0.627
**TC (mmol/L)**	4.72±2.42	4.49±1.26	0.058
**HDL-C (mmol/L)**	1.11±0.36	1.08±0.34	0.262
**LDL-C (mmol/L)**	2.93±0.97	2.96±0.98	0.606
**Blood glucose (mmol/L)**	6.06±2.90	6.54±3.03	0.008*
**SBP(mmHg)**	142.44±20.12	145.20±20.69	0.028*
**DBP(mmHg)**	82.39±11.19	83.48±11.83	0.124

*P < 0.05 was considered statistically significant. Mild Group: NIHSS score ≤ 3; Moderate or severe Group: NIHSS score > 3; TG: total triglyceride, TC: total cholesterol, LDL-C: low-density lipoprotein cholesterol, HDL-C: high-density lipoprotein cholesterol, SBP: systolic blood pressure, DBP: diastolic blood pressure.

### Relationship between carotid plaque and neurological deficit in patients with cerebral infarction

In the non-carotid plaque group, the number of mild cases (*n* = 261) was larger than that of the moderate to severe group (*n* = 115) (*P* < 0.05). Among the moderate to severe patients, the number of cases in carotid artery plaque group (*n* = 339) was larger than that in non-plaque group (*n* = 115) (*P* < 0.05). These differences between the two groups were statistically significant. (Table 3).

**Table 3:** Comparison of carotid plaque incidence between mild and moderate to severe group.

	Mild Group (n = 624)	Moderate to severe Group (n = 454)	χ^2^	*P* value
**Plaque (-)**	261 (69.41%)	115 (30.59%)	31.486	<0.001
**Plaque (+)**	363 (51.71%)	339 (48.29%)		

Plaque (+): carotid plaque group, plaque (−): non-carotid plaque group; Mild Group: NIHSS score ≤ 3; Moderate to severe Group: NIHSS score > 3.

### Multivariate binary logistics regression analysis of the risks of plaque in patients with cerebral infarction

The risk of plaque was analyzed by binary logistic regression, cerebrovascular risk factors including age, TG, TC, LDL-C, hypertension, diabetes mellitus. The risk of plaque in patients over 60 years old was 2.07 times as higher as that in patients below 60 years old (95% CI: 1.59–2.69, *P* = <0.001). Patients with hypercholesterolemia had 1.62 times risks higher than those of no hypercholesterolemia (95% CI: 1.01–2.61, *P* = 0.047). Patients with hypertension had 1.63 times risks higher than those of no hypertension (95% CI: 1.25–2.12, *P* = <0.001). Patients with diabetes mellitus had 1.83 times risks higher than those without diabetes mellitus (95% CI: 1.31–2.55, *P* = <0.001). The risk of moderate to severe cerebral infarction increased by 2.11 times (95% CI: 1.60–2.77, *P* = <0.001) in patients with carotid plaque than those without carotid plaque, and the risk of moderate to severe cerebral infarction in diabetic patients was 1.40 times higher than that of non-diabetic patients. (95% CI: 1.04–1.87, *P* = 0.026). (Table 4).

**Table 4:** Multivariate Logistics regression analysis of the risks of plaque and stroke severity in patients with cerebral infarction.

Risk factor	n	Plaque			Stroke severity		
		Plaque (+) n	OR (95%CI)	*P* value	Moderate to severe cerebral infarction (n)	OR (95%CI)	*P* value
**Age**							
**<60 y**	518	295	1		215	1	
**≥60 y**	560	407	2.07(1.60–2.69)	<0.001*	239	0.90 (0.70–1.16)	0.434
**TG**							
**<1.7mmol/L**	616	387	1		267	1	
**≥1.7mmol/L**	462	315	1.23(0.94–1.61)	0.133	187	0.83 (0.64–1.07)	0.155
**TC**							
**<5.7 mmol/L**	909	575	1		387	1	
**≥5.7 mmol/L**	169	127	1.61(1.00–2.59)	0.049*	67	0.77 (0.51–1.18)	0.234
**HDL-C**							
**<1.8 mmol/L**	1041	677	1		440	1	
**≥1.8 mmol/L**	37	25	1.06(0.51–2.20)	0.886	14	0.79(0.40–1.57)	0.496
**LDL-C**							
**<4.3 mmol/L**	979	629	1		413	1	
**≥4.3 mmol/L**	99	73	1.03(0.57–1.85)	0.932	41	1.07(0.63–1.80)	0.805
**Hypertension**							
**(-)**	392	225	1		152	1	
**(+)**	686	477	1.63(1.25–2.12)	<0.001*	302	1.14(0.88–1.48)	0.330
**Diabetes mellitus**							
**(-)**	824	508	1		329	1	
**(+)**	254	194	1.82(1.30–2.53)	<0.001*	125	1.39 (1.04–1.87)	0.028*
**Plaque**							
**(-)**	376				115	1	
**(+)**	702				339	2.11(1.60–2.77)	<0.001*

*P < 0.05 was considered statistically significant. TG: total triglyceride, TC: total cholesterol, LDL-C: low-density lipoprotein cholesterol, HDL-C: high-density lipoprotein cholesterol, patients with hypertension (+), patients with diabetes mellitus (+).

### Analysis of the relationship between plaque number and risk of moderate to severe cerebral infarction

All the cerebral infarction patients with carotid plaque were further categorized into the following subtypes: single carotid plaque (*n* = 408) and multiple carotid plaques (*n* = 294). After adjustment of age, TC, TG, HDL-C, LDL-C, hypertension and diabetes mellitus, binary logistic regression analysis also revealed that patients with single plaques had 1.82 times more likely to develop moderate to severe cerebral infarction than those without carotid plaque (95% CI: 1.35–2.45, *P* = <0.001), while patients with multiple carotid plaques had 2.41 times higher to get moderate to severe cerebral infarction than those without carotid plaque (95% CI: 1.74–3.32, *P* = <0.001). (Table 5).

**Table 5:** Analysis of the relationship between plaque number and risk of moderate to severe cerebral infarction.

	Adjusted OR (95%CI)	*P* adjusted
**Type of carotid plaque**		
**Non carotid plaque**	1	
**Single carotid plaque**	1.82(1.35–2.45)	<0.001*
**Multiple carotid plaques**	2.41(1.74–3.32)	<0.001*

*P* adjusted: assessed by logistic regression; adjusted for age, total triglyceride, total cholesterol, low-density lipoprotein cholesterol, high-density lipoprotein cholesterol, hypertension and diabetes mellitus.

*P < 0.05, significant difference.

### Analysis of the relationship of location of infarction between mild and moderate to severe groups

The location of cerebral infarction was classified by one experienced stroke physician according to CT or MRI examinations. Anterior circulation stroke (ACS) was defined as symptomatic ischemia in the territory of the anterior, internal carotid, or middle cerebral arteries. Posterior circulation stroke (PCS) was defined as symptomatic ischemia in the vascular territory of the basilar, vertebral, or the posterior cerebral arteries. In posterior circulation stroke or anterior circulation stroke patients, the number had no statistically significant differences between mild and moderate to severe groups. (Table 6).

**Table 6:** Analysis of the relationship between different severity of cerebral infarction and location of infarction.

	Mild Group (n = 624)	Moderate to severe Group (n = 454)	χ^2^	*P*-value
**Anterior circulation**	439 (57.76%)	321 (42.24%)	0.016	0.900
**Posterior circulation**	185 (58.18%)	133 (41.82%)		

*P < 0.05 was considered statistically significant. Mild Group: NIHSS score ≤ 3; Moderate to severe Group: NIHSS score > 3.

## Discussion

This is the first study to investigate the correlation between carotid atherosclerotic plaques and the severity of cerebral infarction. Our novel finding was obtained as follows: carotid plaque may be related to the degree of neural function defect in patients with cerebral infarction.

This study showed that age, and several known cerebrovascular risk factors such as LDL-C, blood glucose and SBP were higher in plaque group than in non-plaque group. The risk of plaque in patients over 60 years old was 2.07 times as higher as that in patients below 60 years old. The main risk factors for the formation of carotid atherosclerotic plaques include age, hypertension, diabetes, hyperlipidemia, and so on[34]. Long term hypertension can cause changes in vascular wall tension and shear stress, intimal injury, hyperlipidemia and accumulation of lipoprotein; long term hyperglycemia causes vascular wall junction[35]. These factors affect and interact with each other, which can lead to endothelial cell dysfunction, leading to inflammatory fibrous hyperplasia, forming atherosclerotic plaques[36,37].

As expected, our findings are in concordance with those of previous studies [7,38]. Blood glucose and SBP were significantly different between mild and moderate to severe groups, and the risk of moderate to severe cerebral infarction in diabetic patients was 1.40 times higher than that of non-diabetic patients. Therefore, regular detection of traditional cerebrovascular factors such as SBP, LDL-C and blood glucose are conducive to the prevention of atherosclerosis, and particularly useful for identifying cerebral infarction with severe neurological deficit.

The important new findings of this study were that the number of moderate to severe cases in carotid plaque group (*n* = 339) was larger than that in non-plaque group (*n* = 115). At the same time, logistics regression analysis showed that the risk of moderate to severe cerebral infarction increased by 2.11 times with carotid plaque in patients than those without carotid plaque. Binary logistic regression analysis also revealed that patients with single plaques had 1.82 times more likely to develop moderate to severe cerebral infarction than those without carotid plaque, while patients with multiple carotid plaques had 2.41 times higher to get moderate or severe cerebral infarction than those without carotid plaque.

Based on these data it is obvious that the incidence of carotid atherosclerotic plaques was associated with neurological deficits in patients with acute cerebral infarction. However, one previous study, which were performed only on 103 patients, showed no significant relationship between the contribution of coexisting carotid atherosclerosis and early neurological progression[39]. The reason for the different result may be due to the different sample size. In our study, we examine these associations with a larger sample size.

This study also revealed that in posterior circulation stroke or anterior circulation stroke patients, no statistically significant differences were found between mild and moderate to severe groups. The factors that affect the neural function defect of cerebral infarction are complex, such as the type of cerebral infarction, the size of thrombus, the position and type of cerebral infarction, the state of collateral circulation, the internal and external environment of the brain, and the regulating function of the local cerebral blood flow[38]. An early arterial study already confirmed that steno-occlusive arterial disease may have on the development of early neurological deterioration in patients with acute ischemic stroke[40]. The study found that collateral circulation varies dramatically between individuals, and collateral extent is significant predictor of stroke severity and recanalization rate[41]. Alawneh, J.A et al.[42] review the evidence from the literature for a role of hemodynamic and perfusion abnormalities, more specifically infarction of the oligemia. The evidence explained that hemodynamic factors and perfusion abnormalities are likely to play a critical role in early neurological deterioration. These studies have confirmed that the cerebrovascular lesion is an independent risk factor for early neurological deterioration.

The pathological basis of these cerebrovascular lesions is atherosclerosis, which is a chronic and progressive non inflammatory, degenerative and proliferative disease involving the blood vessels of the whole body[34]. It can cause vascular injury remodeling, inflammation, thrombosis, and plaque rupture. The pathological process of atherosclerosis may be divided into three stages: vascular endothelial dysfunction, intima thickening, and atherosclerotic plaque formation [34,43]. Finally, it causes cerebral vascular events [44,45]. Carotid atherosclerotic plaque is widely regarded as a window of systemic atherosclerotic disease. It is easy to observe the changes of intima and hemodynamics by high frequency color Doppler ultrasound, which is based on the superficial carotid position and small disturbance. Jung, K.W., et al reported that a comprehensive assessment of carotid status through noninvasive ultrasonography would be useful for identifying carotid plaques or IMT, which can be underestimated by CT or MR angiography [39]. Therefore, carotid plaques which can provide precise information regarding early or minimal atherosclerotic change in stroke patients before it advances [10,46], associated with neurological deficits in patients with acute cerebral infarction in our study.

There are some limitations in this study. First, the main limitation of this study is this is a cross-section study, which generally does not conduct causal analysis. However, the cross-sectional design can find high-risk groups or related etiology clues through the investigation of a certain area or population, combined with the existing clinical research, so as to provide basis for disease prevention and treatment. In order to further confirm the correlation between neurological deficit and carotid atherosclerotic plaque in patients with cerebral infarction, a prospective follow-up study may demonstrate the facts more clearly. Second, investigating whether plaque composition is related to severity of cerebral infarction would be important. However, histopathological evaluation by ultrasonography showed no significant relationship between the different plaque components while the differences between the observers were significant [47,48]. Therefore, the gray-scale median which is more quantitative and with less inter-observer variability was introduced to characterize the plaque [48,49]. Follow-up studies are warranted to assess the prognostic value of MR imaging-assessed plaque composition with regard to the severity of new cerebral infarcts. Third, personal experience and subjective factors may influence the final outcome of carotid color ultrasound. Nevertheless, because our sample size is large enough, the results are reliable.

In summary, cerebral infarction is a disease with multiple risk factors. So far, there is no good prediction method for the severity of cerebral infarction. This was the first study to show that carotid atherosclerotic plaque in patients with acute cerebral infarction may be correlated with the degree of neural function defect. This study provided a reliable clinical basis for the treatment of cerebral infarction to fully understand the relationship between carotid atherosclerotic plaque and cerebral infarction and early intervention treatment, which is of clinical significance for the prevention and treatment of cerebral infarction.

## Supporting information

S1 STROBE Checklist STROBE statement—Checklist of items that should be included in reports of observational studies. (DOCX)
